# How Much Chronic Disease Out-of-Pocket Expenditure Runs Through Pain? A Prospective Counterfactual Decomposition of Five-Year Korean Panel Data

**DOI:** 10.3390/medicina62081549

**Published:** 2026-08-12

**Authors:** Hangaram Kim, Seungpyo Nam, Beomil Park, Kaehong Lee, Seungcheol Yu, Jeongsoo Kim, Yongjae Yoo, Jee Youn Moon

**Affiliations:** 1 Department of Anesthesiology and Pain Medicine, SMG-SNU Boramae Medical Center, Seoul 07061, Republic of Korea; kmlass@naver.com (H.K.); qjadlf216@naver.com (B.P.); 2 Department of Anesthesiology and Pain Medicine, Seoul National University Hospital, Seoul 03080, Republic of Korea; gspnam@gmail.com (S.N.); carrot809@gmail.com (K.L.); ysc3376@naver.com (S.Y.); dreamsu4@snu.ac.kr (J.K.);; 3 Department of Anesthesiology and Pain Medicine, Seoul National University College of Medicine, Seoul 03080, Republic of Korea

**Keywords:** chronic disease, pain, out-of-pocket payments, exploratory mediation, counterfactual decomposition, g-computation, Korea Health Panel Survey

## Abstract

*Background and Objectives*: Pain has been proposed as a pathway linking chronic disease to healthcare spending, but existing estimates measure exposure, pain and cost in the same period and combine coefficients on incompatible scales. We asked how much of the disease–expenditure association runs through pain when exposure, pain and cost are separated in time. *Materials and Methods*: In the Korea Health Panel Survey (2019–2023; 54,845 adult person-years), chronic disease at year *t*, EQ-5D pain/discomfort at *t* + 1 and out-of-pocket payments during *t* + 2 were linked, conditioning on pain and payments at *t*. Direct and indirect effects were estimated by parametric g-computation (randomised-interventional analogues, two-part outcome model, exposure-specific adjustment sets, survey and censoring weights, false discovery rate control). *Results*: Among 25,692 triplets, pain/discomfort predicted the amount spent among healthcare users (cost ratio, CR 1.099, 95% confidence interval, CI 1.023–1.180) but showed no detectable association with whether care was used (odds ratio, OR 1.136, 0.918–1.406); the contemporaneous cost ratio was substantially larger (1.296), inflated by simultaneity. Three musculoskeletal conditions prospectively predicted pain (false discovery rate q < 0.001). Six conditions had indirect-effect intervals excluding zero, but none survived false discovery rate correction (minimum q = 0.19); where estimable, the proportion mediated was small (2.8% to 4.8%). *Conclusions*: The disease → pain and pain → payment associations are each established, but the disease-specific mediated amount is not confirmed after multiplicity correction. Contemporaneous designs overstate the indirect association; claims that pain causes a specified share of chronic disease expenditure are not supported.

## 1. Introduction

Chronic diseases account for a growing share of healthcare spending in ageing populations [1,2]. In South Korea, where adults aged 65 years and over are projected to exceed 20% of the population, the management of hypertension, diabetes, cancer and musculoskeletal disease represents a rising burden on the National Health Insurance system [3]. Identifying the routes by which individual conditions are associated with costs is a prerequisite for allocating resources efficiently within universal coverage [4,5,6].

Pain is among the most frequently reported symptoms in chronic disease. Roughly one in five adults experiences clinically significant pain at any time, and the proportion rises steeply with age and multimorbidity [7,8,9]. Although pain has been studied predominantly in musculoskeletal conditions, it is also common in diabetes, cerebrovascular disease, cancer and chronic kidney disease [10,11,12,13]. Pain is a common reason for care-seeking and is associated with outpatient visits, analgesic prescriptions, imaging and specialist referral [14]. It has therefore been proposed as an intermediate variable linking chronic disease to expenditure [15,16].

Evidence for this pathway is weaker than its intuitive appeal suggests. Most studies estimate the association between pain and costs without decomposing it. Those that attempt decomposition face two problems that are rarely addressed. First, exposure, pain and cost are typically measured over the same period, so annual expenditure includes the very encounters at which pain and diagnosis were recorded; the direction of the association cannot be established. Second, when a binary mediator is modelled by logistic regression and a skewed cost outcome by a generalised linear model with a log link, the product of the two coefficients is not defined on any interpretable scale, and the ratio formed from it is not a proportion of a total effect [17,18].

We addressed both problems directly. Chronic disease status, pain/discomfort and out-of-pocket payments were separated in time by one year each, and direct and indirect contributions were estimated by parametric g-computation [19], which places every quantity on a single additive scale in Korean won and makes the decomposition exactly additive.

Three questions were addressed. Which chronic conditions prospectively predict pain/discomfort one year later? Does pain/discomfort predict subsequent out-of-pocket payments once prior pain and prior payments are conditioned on? And when the disease–payment association is decomposed on a common scale, how much runs through pain?

We describe the study as exploratory. The design establishes temporal order but not causal identification: residual confounding cannot be excluded, and the EQ-5D pain item cannot attribute a reported symptom to any specific condition. We therefore report a decomposition, not a causal effect, and make no claim that intervening on pain would change spending.

## 2. Materials and Methods

### 2.1. Ethical Considerations

This study used data from the Korea Health Panel Survey (KHPS), a publicly available dataset provided by the Korea Institute for Health and Social Affairs and the National Health Insurance Service. All personally identifiable information had been removed prior to analysis. The survey was reviewed and approved annually by the Institutional Review Board (IRB) of the Korea Institute for Health and Social Affairs (IRB No. 2025-0446). The study protocol was also exempted from review by the IRB of SMG-SNU Boramae Hospital (IRB No. 07-2026-07), in accordance with the Declaration of Helsinki.

### 2.2. Data Sources and Study Population

We used the second phase of the KHPS, covering 2019–2023. The survey employs two-stage stratified cluster sampling based on the 2016 Population and Housing Census: 708 primary sampling units were drawn across 17 provinces stratified by urban and rural residence, and approximately 8500 households were then systematically sampled.

**Figure 1:**
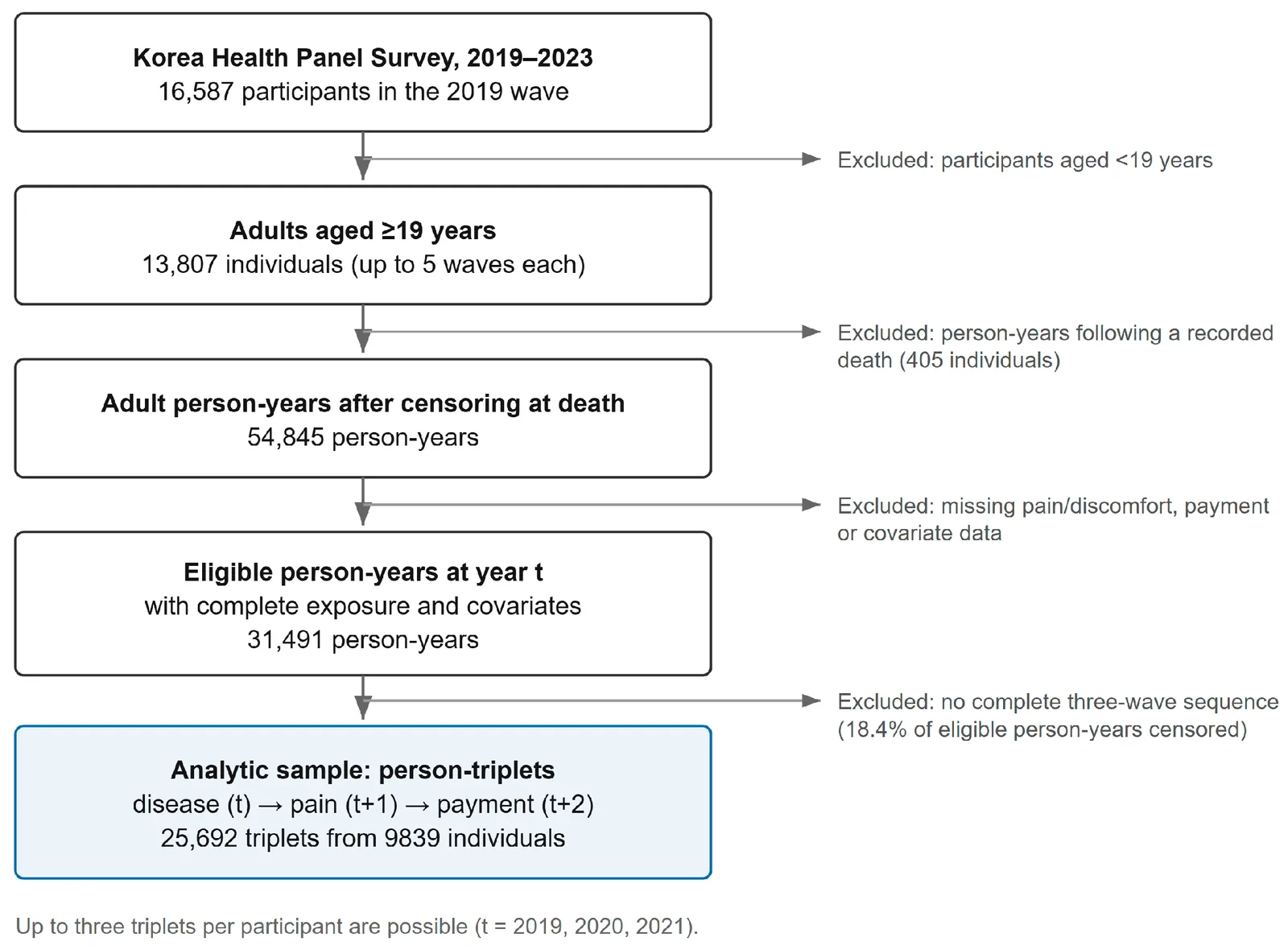
Formation of the person-triplet analytic sample.

The individual files contain 16,587 participants in 2019, declining to 12,169 by 2023. Restricting to adults aged 19 years or older and censoring person-years following a recorded death yielded an analytic base of 54,845 person-years on 13,807 individuals (Figure 1).

### 2.3. Design and Temporal Structure

The unit of analysis is a person-triplet spanning three consecutive waves:
ElementTimingChronic disease status (exposure)year *t*Pain/discomfort (intermediate)year *t* + 1Out-of-pocket payments (outcome)calendar year *t* + 2Covariatesyear *t*Prior pain, prior payments (conditioned on)year *t*

With five waves, t takes values 2019, 2020 and 2021, giving up to three triplets per participant. Conditioning on pain and log payments at t means the analysis asks whether disease status predicts a change in pain, and whether that change predicts subsequent spending, rather than whether these quantities co-occur. Two consequences follow. First, the mediated quantity is confined to pain that changes between t and t + 1: any contribution running through pain already present at t is removed by design, so the proportions mediated reported below are conservative with respect to a pathway through pain at any level. Second, pain at t is itself a consequence of long-standing disease and of unmeasured severity, so conditioning on it may open a path through those unmeasured determinants.

This structure addresses the principal limitation of contemporaneous designs, in which expenditure for year t may include the consultations at which both the chronic condition and the pain were ascertained. To quantify how much that matters, we also fitted the contemporaneous specification and report both.

Temporal ordering does not by itself confer causal identification. Unmeasured common causes of pain and spending—disease severity, treatment intensity, care-seeking propensity—remain possible, and are discussed in Section 4.4.

### 2.4. Variables

#### 2.4.1. Outcome: Out-of-Pocket Medical Payments

The outcome is the participant’s own annual payment, constructed from the KHPS variables EROOP (emergency), INOOP (inpatient), OUOOP_1_MED (medical outpatient) and OUOOP_2_MED (outpatient pharmacy dispensing), each recorded in Korean won. All are amounts settled by the patient so the outcome is out-of-pocket payment, not total charges and not insurer-reimbursed amounts. Values are complete for adults responding to a wave; a missing component denotes non-use of that service and is treated as zero.

Two departures from prior work are deliberate. Outpatient pharmacy payments are included: emergency and inpatient figures already bundle their pharmacy costs, whereas the outpatient figure does not, and analgesic prescribing is a principal channel linking pain to spending. Dental payments are excluded: they are large (mean 193,340 KRW, US$206, among users), driven by implants and prosthetics, and largely unrelated to the chronic conditions under study.

The mean annual payment among adults was 874,760 KRW (US$930), median 370,300 KRW (US$394), with 12.0% of person-years recording no payment. An alternative, narrower definition was examined as a sensitivity analysis (Supplementary Table S3).

Because Korea’s National Health Insurance covers approximately two-thirds of medical costs, out-of-pocket amounts are small in absolute terms and poorly comparable internationally. The results are therefore presented primarily as cost ratios and proportions mediated, with won amounts retained alongside.

To orient readers unfamiliar with the currency, won amounts are accompanied throughout by an approximate equivalent in purchasing-power-parity-adjusted US dollars, written US$ (PPP), using the World Bank purchasing-power-parity conversion factor for private consumption. The factor is matched to the year of measurement: 984.1 KRW per US$ for the 2019 baseline characteristics of Table 1, 940.7 for descriptive amounts drawn from the whole 2019–2023 base, and 916.0—the mean of the three outcome years 2021–2023 (961.0, 904.0 and 883.1)—for the decomposition results, whose outcome is measured at t + 2. These are not market-rate conversions: the market exchange rate averaged 1165.4 KRW per US$ in 2019 and 1247.0 over 2021–2023, so market-rate figures would be between about 16% and 27% smaller than the parity-adjusted ones quoted here.

#### 2.4.2. Intermediate Variable: Pain/Discomfort

Pain/discomfort was measured by the EQ-5D pain/discomfort dimension (HS_EQ4; 1 = none, 2 = moderate, 3 = extreme) and dichotomised as present (≥2) versus absent, the proportional odds assumption being violated. We use the term “pain/discomfort” throughout: the item has a same-day reference period, includes discomfort as well as pain, and provides no information on duration, intensity or site. It cannot establish that a reported symptom arises from the participant’s chronic condition [20]—a limitation with direct bearing on interpretation (Section 4.4).

#### 2.4.3. Exposures: Chronic Conditions

Conditions were physician-diagnosed and recorded as ever-diagnosed, coded from the KHPS chronic disease block. Spine disorders combine disc disorder and other spine disease; the KHPS revised these items in 2021, and although the subcategories are not comparable across that boundary the composite is, with within-person transition rates of 2.77%, 2.49%, 2.30% and 1.83% for consecutive year pairs and no discontinuity at 2020–2021. Subcategory analysis of spine disease is therefore not reported.

Shoulder disorders were excluded: the item was introduced only in 2021 and is unavailable for the first two waves. Conditions with fewer than 50 exposed individuals were excluded from modelling and are reported descriptively. Incident cases were identified as the first within-person transition from absent to present; because this requires a prior wave, incident status is defined only from 2020 (Section 3.6).

#### 2.4.4. Covariates and Confounder Selection

Covariates measured at year t comprised age, sex, marital status, education (six categories, indicator-coded, high school as reference), equivalised household income (square-root scale), disability, urban residence, height, weight, regular exercise, days walked per week, lifetime smoking (three categories), and depressive mood, anxiety and suicidal ideation in the past year [21,22,23].

A single adjustment set applied to every condition is not defensible when the conditions are causally related to one another: adjusting the hypertension model for stroke, chronic kidney disease or myocardial infarction conditions on consequences of hypertension rather than on confounders. We therefore derived exposure-specific adjustment sets from a directed acyclic graph (DAG) specified a priori, before estimation, on clinical grounds (Supplementary Figure S1, Supplementary Table S5, which cites the sequential relationships used). For each index condition, established downstream sequelae were excluded from its covariate set—for example, hypertension is not adjusted for chronic kidney disease, stroke, myocardial infarction, angina or dementia; rheumatoid arthritis is not adjusted for secondary osteoarthritis; thyroid cancer is not adjusted for post-surgical hypothyroidism; and conditions that generate pain are not adjusted for physician-diagnosed depression.

Physician-diagnosed depression or bipolar disorder is one of the exposures, while depressive mood, anxiety and suicidal ideation are covariates; the two overlap conceptually, and the sensitivity analysis omitting the mental-health covariates (Section 3.5, Supplementary Table S8) bears directly on that overlap. Self-rated health was excluded. It is a downstream consequence of both chronic disease and pain and is associated with care-seeking, so conditioning on it would block part of the association under study and, as a common effect of the exposure and of unmeasured determinants of spending, would open a collider path.

### 2.5. Missing Data, Censoring and Weighting

Among adults responding to a wave, out-of-pocket payments have no item missingness. The EQ-5D health module was not administered to 7.5% of adult respondents; because the module is fielded as a block, all module variables share that rate. The dominant source of incomplete data is wave non-response.

The analysis is complete-case with respect to pain and payments. We did not impute whole unobserved waves: pain is a subjective state that adjacent-wave information predicts imperfectly, and 21% of dementia person-years lack pain data because the module was completed by proxy—imputing a self-reported symptom for participants who could not report it is not defensible. Dementia is therefore excluded from interpretation.

Person-years following a recorded death (DEATH_I_YN; 405 individuals) were censored rather than treated as missing.

Two weights were applied multiplicatively. The KHPS cross-sectional individual weight I_WGC, complete in every wave, accounts for unequal selection probability and initial non-response. Attrition was addressed by stabilised inverse-probability-of-censoring weights (IPCW) [24], estimated from a logistic model for contributing a complete triplet given all conditions, covariates, prior pain and prior log payments at t. Among 31,491 eligible person-years, 81.6% contributed a complete triplet. Censoring was informative. Odds ratios for contributing a complete triplet were 0.28 for dementia, 1.06 per unit of prior log payment, 1.34 per standard deviation of age and 1.37 for private health insurance: participants with dementia were markedly less likely to be retained, while older, higher-spending and privately insured participants were more likely to be retained. Weights were well behaved (mean 1.000, SD 0.111, range 0.82–3.21) and trimmed at the 1st and 99th percentiles.

Stratum and primary sampling unit identifiers are not distributed with the individual-level KHPS files and were not available for this analysis. Standard errors therefore derive from a participant-level bootstrap, which accounts for repeated measures within individuals but not for the clustering induced by the original two-stage sampling design. Confidence intervals may consequently be somewhat narrower than a fully design-based analysis would produce; this affects the precision of the estimates rather than their magnitude, and bears most on findings whose intervals lie close to the null.

### 2.6. Statistical Analysis

#### 2.6.1. Estimand

We estimate randomised-interventional analogues of the direct and indirect effects [25,26], not natural effects. The natural indirect effect requires cross-world independence, which is not credible here, and is not identified when mediator–outcome confounders are themselves affected by the exposure—disease severity and treatment intensity being obvious candidates. The interventional analogue requires neither and remains well defined under both [25,27]. The scope of this choice should be stated plainly: exposure-induced mediator–outcome confounders such as disease severity and treatment intensity are not measured in the KHPS and so do not enter estimation. The interventional formulation therefore secures an estimand that remains well defined in their presence; it does not adjust for them, and its advantage over the natural decomposition here is one of definition rather than of identification. The estimand and the g-computation algorithm are stated formally in Supplementary Text S1.

Both components are differences in expected out-of-pocket payment, in won, so that total = direct + indirect exactly. Given the small absolute magnitudes typical of a high-coverage insurance system, we also express the total effect as a cost ratio relative to the unexposed counterfactual, and the indirect component as a proportion of the total.

#### 2.6.2. Models and Estimation

Three models were fitted for each condition: a logistic model for pain/discomfort at t + 1, fitted by generalised estimating equations with an exchangeable working correlation [28,29], and a two-part outcome model for payments at t + 2, comprising a logistic model for any payment and a gamma model with log link for the amount among those with positive payments. The two-part specification is required because 12% of person-years record no payment; a gamma-only model would change the estimand to spending conditional on being a healthcare user [30]. A gamma model with a log link is used for the positive part because health expenditure is right-skewed and heteroscedastic on the log scale [31]. Survey year enters every model as a categorical term rather than as a linear one, so that inflation, policy change and the non-linear course of the COVID-19 pandemic are absorbed without a functional-form assumption; for the same reason, and because the outcome is reported primarily as a ratio, no conversion to a common price year is applied. Zero-payment person-years are reported by wave in Supplementary Table S1, and fall from 16.8% in 2019 to 7.5% in 2023.

Expected payment under each combination of exposure and mediator was computed by summing over the binary mediator distribution analytically, avoiding Monte Carlo error. Confidence intervals come from 2000 participant-level bootstrap replicates, resampling whole individuals to preserve within-person correlation; total, direct and indirect effects were retained for every replicate so that intervals for the cost ratio and the proportion mediated are obtained directly rather than by approximation.

#### 2.6.3. Multiplicity and Reporting Rules

Benjamini–Hochberg false discovery rate control [32] at 5% was applied twice, in each case across the 22 modelled conditions: once to the disease → pain coefficients of the mediator model, and once to the disease-specific indirect effects of the decomposition. Both applications were specified before the results were inspected. Because the indirect effect has no closed-form standard error, a standard error was derived from the width of its bootstrap percentile interval and a two-sided normal-approximation *p*-value formed from it; these *p*-values entered the Benjamini–Hochberg procedure. Two criteria are therefore in use and they are not interchangeable. A condition is described as showing an indirect signal when its bootstrap percentile interval excludes zero, and as surviving or failing correction on the basis of its q-value. Where the bootstrap distribution is skewed the two need not agree: for rheumatoid arthritis, asthma and angina pectoris the percentile interval excludes zero while the approximate *p*-value is 0.067, 0.097 and 0.106 respectively. Both are reported for every condition in Supplementary Table S2, and q-values are reported alongside the intervals throughout.

The proportion mediated is reported only where the bootstrap confidence interval for the total effect excludes zero. This rule is applied programmatically. It matters because the proportion inherits the instability of its denominator: for non-knee osteoarthritis the indirect effect is 8881 KRW (US$9.70) under one expenditure definition and 8539 KRW (US$9.32) under another—a 4% difference—while the proportion moves from 27.2% to 82.0% because the total effect differs. Reporting such percentages without guarding the denominator would repeat, on a different scale, the error this reanalysis was undertaken to correct.

#### 2.6.4. Pre-Specified Sensitivity Analyses

Pre-specified: an alternative expenditure definition; the contemporaneous specification for comparison; with and without IPCW; incident versus prevalent disease; and, given the reviewers’ observation that contemporaneous mental-health variables may be consequences rather than confounders, a specification omitting them.

### 2.7. Software

Analyses were conducted in Python 3.11 (Python Software Foundation, Wilmington, DE, USA) using statsmodels 0.14, pandas, numpy and patsy. A generative artificial intelligence tool (Gemini 3.1 Pro; Google LLC, Mountain View, CA, USA) assisted with coding for data visualisation and with language editing; all output was reviewed and verified by the authors, who take full responsibility for the content.

## 3. Results

### 3.1. Study Population

The analytic base comprised 54,845 adult person-years on 13,807 individuals. At baseline (2019), 11,593 adults had both pain/discomfort and payments observed; 27.8% (survey-weighted) reported pain/discomfort. The decomposition was fitted on 25,692 person-triplets contributed by 9839 individuals (Figure 1).

Participants reporting pain/discomfort were older (57.6 vs. 46.6 years; standardised mean difference 0.72), more often female (62.1% vs. 50.6%; SMD 0.23), less educated (mean category 3.38 vs. 2.61 on a scale where 1 denotes graduate education and 6 no formal schooling; SMD 0.63), and far more likely to have a registered disability (9.7% vs. 2.4%; SMD 0.31). They reported more depressive mood (14.6% vs. 6.0%), anxiety (9.5% vs. 3.7%) and suicidal ideation (14.0% vs. 4.6%), and had lower equivalised household income (24.4 vs. 32.7 million KRW, US$24,865 vs. US$33,289).

Median annual out-of-pocket payment was 471,215 KRW (IQR 156,230–1,067,692), about US$479 (US$159–1085), in the pain/discomfort group and 192,680 KRW (20,700–551,675), about US$196 (US$21.0–561), in its absence; 8.2% and 19.4% respectively recorded no payment. Full characteristics appear in Table 1.

### 3.2. Chronic Disease and Subsequent Pain/Discomfort

In the mediator model—all 22 conditions entered simultaneously, disease at t predicting pain/discomfort at t + 1, conditioning on pain at t—three conditions survived false discovery rate control:
ConditionOR (95% CI)qSpine disorders1.751 (1.561–1.964)<0.001Osteoarthritis, non-knee1.733 (1.430–2.101)<0.001Knee osteoarthritis1.552 (1.382–1.744)<0.001

Rheumatoid arthritis (OR 1.668, q = 0.065), angina pectoris (1.342, q = 0.065) and asthma (1.487, q = 0.076) approached but did not cross the threshold. Prior pain/discomfort was by far the strongest predictor (OR 2.972, 95% CI 2.690–3.285; Table 2), confirming that the item is highly autocorrelated within individuals and underlining the importance of conditioning on it. A corollary is that, once prior pain is conditioned on, the residual variation in pain that disease status can explain is modest, which limits statistical power and is one reason few conditions reach significance.

No cardiovascular, metabolic, renal, hepatic or oncological condition predicted subsequent pain/discomfort after adjustment. Complete results appear in Table 2.

### 3.3. Pain/Discomfort and Subsequent Payments

Pain/discomfort at t + 1 did not predict whether any care was used in t + 2 (OR 1.136, 95% CI 0.918–1.406, *p* = 0.24), an interval wide enough to remain compatible with a moderate association. It did predict the amount spent among those who used care—an intensive-margin effect only—with a cost ratio of 1.099 (95% CI 1.023–1.180, *p* = 0.010).

**Figure 2:**
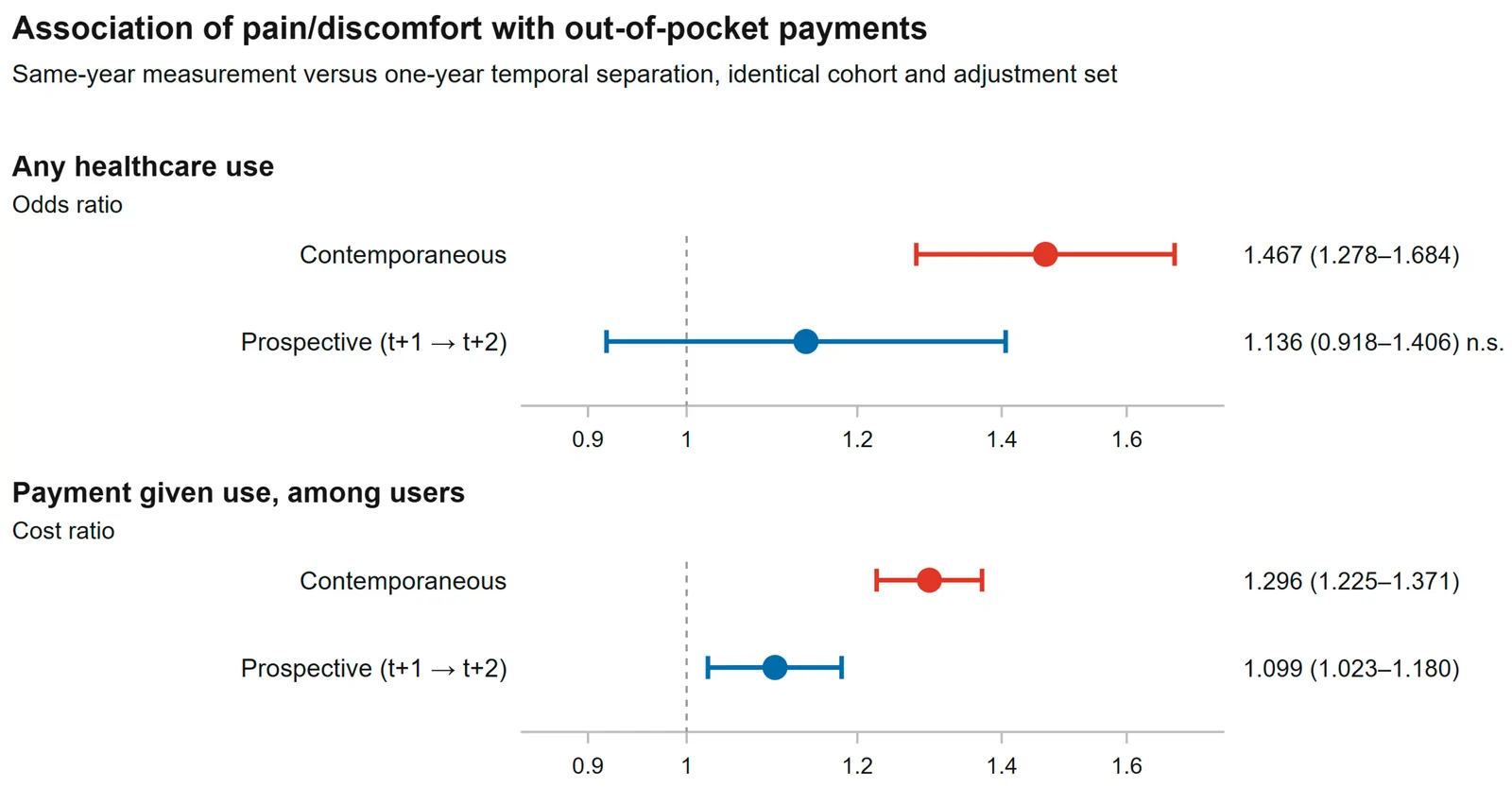
Contemporaneous versus prospective estimates of the pain–payment association. Both specifications are fitted in the same cohort with the same adjustment set, conditioning on pain and payments at year t, and differ only in the timing of measurement. Neither estimate should be read as correct on its own: the contemporaneous estimate is biased upwards by simultaneity, because a year’s expenditure includes the consultations at which pain and diagnosis were recorded, while the prospective estimate is biased downwards because it omits pain arising and resolving between annual surveys, 40% of reported pain having resolved within one year (Supplementary Table S9). The two are therefore expected to be biased in opposite directions; this does not establish that the true value lies between them. Under temporal ordering the association with whether any care was used is no longer detectable, whereas the association with the amount spent among users persists. All models adjust for prior pain, prior payments, all chronic conditions and the full covariate set, and apply survey weights. CI, confidence interval; n.s., 95% CI includes 1.

The contrast with the contemporaneous specification is the central methodological finding of this study (Figure 2):
ComponentContemporaneousProspective (*t* + 1 → *t* + 2)Any healthcare useOR 1.467 (1.278–1.684)OR 1.136 (0.918–1.406)Payment given useCR 1.296 (1.225–1.371)CR 1.099 (1.023–1.180)

The care-seeking component disappears entirely once pain must precede spending and prior spending is conditioned on. The amount component falls substantially but persists. Part of this difference is an artefact of simultaneity—the year’s expenditure includes the encounters at which pain was recorded, so the contemporaneous estimate is inflated by reverse timing. Part of it, however, reflects a genuine limitation of the prospective design. Pain/discomfort is a highly transient state: of participants reporting pain/discomfort in one wave, 40% no longer reported it one year later (Supplementary Table S9). Because the EQ-5D item refers to the survey day and waves are one year apart, the prospective analysis captures only pain that persists across the interval and misses short-lived or intermittent pain together with the expenditure associated with it. The contemporaneous and prospective estimates are therefore biased in opposite directions by identifiable mechanisms: the contemporaneous cost ratio (1.296) is inflated by simultaneity and probably overstates the effect, while the prospective cost ratio (1.099) omits transient pain and probably understates it. We do not claim they are exact bounds—residual confounding could act in either direction—and we make no claim that the true value lies between them. All decomposition results below use the prospective estimate and are consequently conservative.

### 3.4. Counterfactual Decomposition

The counterfactual decomposition is reported in Table 3. The decomposition models are fitted one condition at a time with exposure-specific adjustment sets (Section 2.4.4), and so differ from the single simultaneous mediator model of Table 2; path-A estimates are correspondingly not identical between the two.

Three overlapping sets of conditions arise and are worth distinguishing: three conditions predict pain after correction (spine disorders, knee and non-knee osteoarthritis; Section 3.2); six have a per-condition indirect-effect interval excluding zero (those three plus rheumatoid arthritis, asthma and angina pectoris); and among those six, three permit an interpretable proportion mediated, because only for those three does the confidence interval for the total effect exclude zero (spine disorders, knee osteoarthritis and angina pectoris; Table 3). Of those three, two also survived correction in the mediator model. However, when the false discovery rate correction applied to the mediator model was extended to the 22 indirect-effect tests, none remained significant (minimum q = 0.19). The disease-specific mediated amounts are therefore hypothesis-generating rather than confirmatory, and we do not claim a confirmed pathway for any individual condition. This loss of significance under correction reflects the limited precision of 22 simultaneously tested indirect effects, not a demonstrated absence of an effect.

Two conditions were the most consistent across every criterion. Spine disorders and knee osteoarthritis each survived false discovery rate control in the mediator model (Section 3.2), each had a total effect whose interval excluded zero—permitting a proportion mediated of 4.8% (95% CI 1.1–10.2) and 4.3% (1.0–10.1) respectively—and each was robust to censoring weights and to the expenditure definition. For three further conditions (non-knee osteoarthritis, rheumatoid arthritis, asthma) the total effect was not distinguishable from zero, so no proportion is defined and only the sign of the indirect component is interpretable; rheumatoid arthritis additionally had a total-effect point estimate below one with only 589 exposed person-years and is reported descriptively. Angina pectoris is the least secure of the set: it did not survive false discovery rate control in the mediator model, its mechanism is cardiac rather than musculoskeletal, and its confidence intervals lie closest to the null.

No other condition—including hypertension, diabetes, chronic kidney disease, cerebrovascular disease and every cancer—showed even a per-condition indirect effect distinguishable from zero; all 22 are reported in Table 3 so that the conditions without a signal are visible alongside those with one.

Both component associations are established, though only the first was subject to multiplicity correction: three musculoskeletal conditions prospectively predict pain/discomfort (Table 2, all q < 0.001), and pain/discomfort prospectively predicts a higher payment among those who use care (cost ratio 1.099, *p* = 0.010, a single pre-specified test outside both correction families; the any-use component is not detectable). These establish the two links from which a pathway would be built, but they are not a substitute for the composed test: the pain → payment estimate is a population average, not specific to pain arising from musculoskeletal disease, and the direct test of the composition—the disease-specific indirect effect—does not survive correction. Whether the composition yields a material disease-specific cost pathway is therefore suggested but not confirmed. Because the indirect effects are estimated imprecisely (wide bootstrap intervals across 22 conditions), their failure to survive correction reflects limited precision rather than demonstrated absence of an effect.

### 3.5. Sensitivity Analyses

**Attrition.** IPCW left conclusions unchanged: the indirect-effect intervals of the same six conditions continued to exclude zero, and the point estimates moved by less than 3% (spine disorders 10,689 KRW → 10,644 KRW (US$11.67 → US$11.62); knee osteoarthritis 8043 KRW → 8075 KRW (US$8.78 → US$8.82); asthma 6333 KRW → 6442 KRW (US$6.91 → US$7.03)). Although censoring was clearly informative, the conditions most affected—dementia and lung cancer—were not among those showing a pathway.

**Expenditure definition.** The indirect-effect intervals of the same six conditions continued to exclude zero under the alternative, narrower definition, with point estimates differing by 1–4% (Supplementary Table S3).

**Mental-health covariates.** Omitting contemporaneous depressive mood, anxiety and suicidal ideation increased indirect effects for most conditions and brought one further condition’s interval to exclude zero, consistent with those variables lying partly on the pathway rather than confounding it (Supplementary Table S8). Primary estimates are therefore conservative in this respect.

### 3.6. Incident Versus Prevalent Disease

Incident cases could be defined only from 2020, reducing the sample to 16,739 triplets and splitting each exposure into two thinner strata. No condition’s indirect-effect interval excluded zero in this analysis. Estimates were directionally consistent with the main results and of similar magnitude—for knee osteoarthritis, 5202 KRW (US$5.68) for incident and 5005 KRW (US$5.46) for prevalent disease—with no systematic difference between newly diagnosed and long-standing disease, but confidence intervals were too wide to support conclusions. Five waves do not provide sufficient incident cases for pathway analysis; this is itself worth recording for the design of future studies.

## 4. Discussion

### 4.1. Summary of Findings

Using five annual waves of nationally representative Korean panel data with one-year separation between exposure, intermediate variable and outcome, we found that three musculoskeletal conditions prospectively predicted pain and that pain prospectively predicted a higher payment among healthcare users; the disease → pain associations survived correction for multiple testing, and the pain → payment association was significant as a single pre-specified test. The composed, disease-specific mediated amounts did not survive correction, so the component associations are established but the composed disease-specific quantity is not confirmed. Where a proportion mediated was estimable, in spine disorders and knee osteoarthritis, it was small (4.8% and 4.3%). Pain/discomfort was not a detectable pathway for the broad range of non-musculoskeletal conditions in which it has been proposed.

The most consequential finding is methodological. In the same data, the contemporaneous association between pain and healthcare utilisation (OR 1.467) was no longer detectable under temporal ordering (OR 1.136, 0.918–1.406). Annual expenditure includes the consultations at which pain and diagnosis are ascertained, so same-year designs cannot separate pain that generates spending from spending that generates a pain record.

### 4.2. Comparison with Previous Studies

The association between pain and healthcare use is well established; chronic pain has been estimated to consume 3–10% of gross domestic product in Europe [33], and Korean studies report higher spending among people with musculoskeletal pain [34,35]. Our results are consistent with those associations while showing that the prospective, decomposed contribution is considerably smaller than cross-sectional estimates imply.

Published mediated proportions in this literature are typically an order of magnitude larger than ours. Three features of our design account for the difference, and all three are corrections rather than conservatism: temporal separation removes simultaneity; a common additive scale removes the incoherence of multiplying log-odds by log-cost-ratio coefficients; and exposure-specific adjustment removes over-adjustment by conditions that are consequences of the exposure. In a diagnostic comparison conducted during the reanalysis, replacing the common adjustment set with exposure-specific sets moved the total effect of diabetes from 558 to 16,641 KRW (US$0.61 to US$18.17) and reduced the implausible negative total effect of hypertension from −157,946 to −141,869 KRW (−US$172 to −US$155); under the final specification, which additionally carries censoring weights, both are positive (188,244 and 60,421 KRW (US$205 and US$66.0) respectively; Supplementary Table S2). The size of these shifts is itself informative: total effects in this setting are sensitive to the adjustment set, and an estimate conditioned on a condition’s own sequelae should not be read at face value.

### 4.3. Why Musculoskeletal Conditions

The conditions that show a signal are those in which pain is the presenting symptom rather than an accompaniment. In osteoarthritis and spine disease, care-seeking is driven by symptom severity rather than radiographic findings [36,37,38], so a pathway through reported pain is mechanistically expected. Asthma and angina both present as somatic discomfort that prompts contact with services.

Cancer spending is likewise dominated by surgery, systemic therapy and surveillance rather than by symptom-driven contact [39]. In hypertension, diabetes and chronic kidney disease, spending arises from monitoring, medication and complications that are managed irrespective of symptoms. Pain may accompany these conditions without lying on the path to their costs. Cross-sectional estimates, including our own earlier analysis of these data, have reported substantial mediated proportions for exactly these conditions; the present analysis does not support them.

### 4.4. Limitations

**Measurement of pain.** The EQ-5D item is binary, refers to the survey day, and cannot attribute a symptom to a condition. A participant with hypertension reporting discomfort may have a musculoskeletal strain or a toothache. Any disease-specific pathway estimate inherits this ambiguity, and it is a principal reason we describe the study as exploratory.

**Transient pain and the one-year lag.** Pain/discomfort is highly transient: 40% of those reporting it in a given wave did not report it one year later (Supplementary Table S9), and pain arising and resolving entirely between two annual surveys is unobserved. Because the outcome is separated from the intermediate variable by a further year, the prospective design captures the expenditure consequences only of pain that persists across the interval, and undercounts short-lived and intermittent pain and its costs. This is the counterpart of the simultaneity that inflates the contemporaneous estimate: the two designs are expected to be biased in opposite directions, with the contemporaneous cost ratio of 1.296 probably overstating and the prospective cost ratio of 1.099 probably understating the effect of persistent pain (Section 3.3). We report the prospective estimate as the primary result because it is the one temporal ordering can defend, and note that it is conservative with respect to acute pain. A design with a shorter recall interval, or linkage to encounter-level claims that timestamp care within the year, would be required to recover the transient component.

**Identification.** Temporal ordering is not identification. Unmeasured determinants of both pain and spending—disease severity, treatment intensity, care-seeking propensity—remain plausible. We estimate interventional analogues, which do not require cross-world independence, but no design feature here rules out confounding of the mediator–outcome relationship. Covariates are moreover fixed at year t; determinants that evolve between t and t + 2, such as household income and mental health, are therefore controlled only at baseline, and any time-varying confounding they introduce is not removed.

**Outcome scope.** The outcome is the patient’s own payment under a system covering roughly two-thirds of costs. It is a measure of household financial burden, not of health-system cost, and no inference about system-level savings follows. Informal costs, over-the-counter medication and caregiving are excluded. Annual totals sum only episodes whose cost the respondent could report, so high-utilisation participants—including those with pain—are undercounted, biasing estimates towards the null.

**Attrition and structural missingness.** Approximately 18% of eligible person-years did not contribute a complete triplet, and censoring was informative. IPCW left results unchanged, but weighting cannot correct for departures from the assumption that censoring is at random given observed history. Dementia is excluded from interpretation on the separate ground that a fifth of its person-years lack self-reported pain.

**Implausible negative total effects.** For several sparse conditions the estimated total effect is negative and of a magnitude that cannot be taken at face value: −146,955 KRW (−US$160) for colorectal cancer, −114,701 (−US$125) for breast cancer and −72,732 (−US$79.4) for rheumatoid arthritis (Supplementary Table S2). We do not read these as evidence that the conditions reduce out-of-pocket payment. Each rests on few participants—66, 78 and 132 respectively in the triplet sample (Supplementary Table S6)—and each requires three consecutive waves of participation, which selects against the severely ill and the recently deceased in precisely those conditions where severity determines cost. Censoring weights address loss to follow-up given observed history but cannot recover participants whose disease trajectory removed them from the panel. These totals are reported for completeness rather than interpreted, and the indirect components attaching to them, rheumatoid arthritis included, should be read with the same reservation.

**Disease definitions.** Conditions are self-reported physician diagnoses without severity, stage or duration, recorded as ever-diagnosed. The spine composite spans a 2021 question redesign; the composite is stable across it but subcategories are not. Shoulder disorders could not be analysed. Incident and prevalent disease could not be distinguished with adequate precision.

**Design variables.** Stratum and primary sampling unit identifiers were unavailable, so variance estimation accounts for repeated measures within participants but not for the two-stage sampling design. Confidence intervals are therefore likely to be somewhat narrow, and the conditions whose intervals lie closest to the null—angina pectoris and asthma in particular—should be regarded as the least secure of the six.

### 4.5. Implications

Pain/discomfort warrants measurement in health-economic models of musculoskeletal disease: it is prospectively associated with subsequent out-of-pocket payments, and the association survives adjustment for the conditions themselves, socioeconomic position, disability and mental health.

It does not follow that treating pain would reduce spending. That inference requires the pathway to be causal, the measure of pain to be attributable to the condition, and the intervention to act on the same quantity the model represents. None is established here. The appropriate next step is a trial or quasi-experiment in which pain treatment is varied and spending observed, not further observational decomposition.

The methodological implication is more immediately actionable. Where exposure, symptom and cost share an observation period, mediated proportions should be regarded as likely overstatements of unknown magnitude. Where a decomposition is attempted, both components must be defined on one scale; a product of coefficients from a logistic and a log-link model is not a quantity with an interpretation.

## 5. Conclusions

In a nationally representative Korean cohort with one-year separation between exposure, pain and cost, two component associations were established: three musculoskeletal conditions prospectively predicted pain/discomfort, surviving correction for multiple testing, and pain/discomfort prospectively predicted a higher out-of-pocket payment among healthcare users (cost ratio 1.099; the any-use component was not detectable). These establish the links of a pathway but not its composed disease-specific magnitude. Six conditions—predominantly musculoskeletal, with asthma and angina pectoris as weaker signals—showed a per-condition indirect effect distinguishable from zero, but none survived false discovery rate correction, so no disease-specific mediated amount can be claimed with confidence. The most consistent conditions were spine disorders and knee osteoarthritis, for which the estimable proportion mediated was 4.8% and 4.3%. No pathway of any kind was detectable for hypertension, diabetes, chronic kidney disease, cerebrovascular disease or any cancer.

Under temporal ordering, pain predicted how much was spent but showed no detectable association with whether care was sought; the contemporaneous association with utilisation was inflated by simultaneity, while the prospective estimate omits pain arising and resolving within the one-year interval and probably understates the effect of persistent pain. Findings were robust to attrition weighting and to the expenditure definition used.

These data are consistent with a modest and condition-specific indirect association between pain/discomfort and household medical spending. They do not support quantitative claims about the share of chronic disease expenditure caused by pain, nor the inference that pain-directed treatment would reduce it.

## Figures and Tables

**Table 1:** Baseline characteristics at 2019 by pain/discomfort status.

Characteristic	Pain/Discomfort (*n* = 3950)	No Pain/Discomfort (*n* = 7643)	SMD
Age, years, mean	57.6	46.6	0.72
Female, %	62.1	50.6	0.23
Married, %	61.6	67.0	−0.11
Education category, mean (1 = graduate school … 6 = none)	3.4	2.6	0.63
Equivalised household income, 10,000 KRW	2447 [US$24,865]	3276 [US$33,289]	−0.45
Urban residence, %	80.5	84.5	−0.11
Registered disability, %	9.7	2.4	0.31
Private health insurance, %	69.9	83.6	−0.33
Height, cm, mean	161.9	165.6	−0.43
Weight, kg, mean	63.0	64.7	−0.15
Regular exercise, %	49.0	53.5	−0.09
Days walked per week, mean	4.0	4.5	−0.20
Lifetime smoking <5 packs, %	1.8	2.5	−0.05
Lifetime smoking ≥5 packs, %	34.0	35.8	−0.04
Depressive mood, %	14.6	6.0	0.29
Anxiety, %	9.5	3.7	0.23
Suicidal ideation, %	14.0	4.6	0.33
Out-of-pocket payment, median, KRW	471,215 [US$479]	192,680 [US$196]	—
Interquartile range	156,230–1,067,692 [US$159–1085]	20,700–551,675 [US$21.0–561]	—
No payment recorded, %	8.2	19.4	—
**Chronic conditions, %**			
Hypertension	33.81	15.84	0.43
Diabetes mellitus	14.82	7.49	0.23
Chronic hepatitis B/C	0.95	0.57	0.04
Liver cirrhosis	0.57	0.20	0.06
Knee osteoarthritis	19.10	2.68	0.55
Osteoarthritis, non-knee	7.71	1.34	0.31
Rheumatoid arthritis	1.86	0.29	0.15
Gastric cancer	0.47	0.37	0.02
Colorectal cancer	0.53	0.16	0.06
Lung cancer	0.34	0.14	0.04
Breast cancer	0.82	0.47	0.04
Cervical cancer	0.28	0.16	0.03
Thyroid cancer	0.99	0.87	0.01
Other cancer	1.76	0.73	0.09
Angina pectoris	2.69	0.90	0.14
Myocardial infarction	2.77	1.22	0.11
Cerebral haemorrhage	1.26	0.27	0.11
Cerebral infarction	3.89	0.91	0.20
Asthma	1.67	0.43	0.12
COPD	0.35	0.13	0.05
Bronchiectasis	0.39	0.17	0.04
Hypothyroidism	2.25	1.22	0.08
Hyperthyroidism	0.88	0.61	0.03
Depression/bipolar disorder	4.71	0.95	0.23
Dementia	0.92	0.13	0.11
Chronic kidney disease	0.75	0.20	0.08
Spine disorders	23.73	3.72	0.61

Values are survey-weighted means or percentages; n are unweighted counts. Weighted prevalence of pain/discomfort at baseline was 27.8%. SMD, standardised mean difference; a value below 0.10 indicates negligible imbalance. Education is coded 1 = graduate school, 2 = university, 3 = high school, 4 = middle school, 5 = elementary school, 6 = no formal schooling, so higher values denote less education. Household income is equivalised by the square root of household size. Lifetime smoking is categorised as never (reference), <5 packs and ≥5 packs. Payments are patient out-of-pocket amounts and exclude dental care. Conditions with fewer than 20 affected participants at baseline are omitted from this table; the threshold for entry into the models was 50 exposed individuals (Section 2.4.3).

**Table 2:** Chronic disease at year t and pain/discomfort at year t + 1: (**A**) chronic conditions and (**B**) covariates, from a single generalised estimating equation model.

**A. Chronic Conditions.**					
**Condition**	**Odds Ratio**	**95% CI**	***p***	***q* ** **(FDR)**	**Significant at 5% FDR**
Spine disorders	1.751	1.561–1.964	<0.0001	<0.001	Yes
Knee osteoarthritis	1.552	1.382–1.744	<0.0001	<0.001	Yes
Osteoarthritis, non-knee	1.733	1.430–2.101	<0.0001	<0.001	Yes
Rheumatoid arthritis	1.668	1.108–2.510	0.0142	0.065	
Angina pectoris	1.342	1.059–1.700	0.0147	0.065	
Asthma	1.487	1.063–2.082	0.0206	0.076	
Depression/bipolar disorder	1.255	0.949–1.661	0.111	0.350	
Breast cancer	1.288	0.923–1.796	0.136	0.375	
Cerebral infarction	1.174	0.911–1.512	0.215	0.479	
Cerebral haemorrhage	1.359	0.834–2.214	0.218	0.479	
Colorectal cancer	0.801	0.536–1.198	0.281	0.561	
Thyroid cancer	1.207	0.802–1.816	0.368	0.663	
Chronic kidney disease	1.267	0.737–2.178	0.392	0.663	
Diabetes mellitus	1.046	0.929–1.177	0.459	0.681	
Hypothyroidism	1.107	0.843–1.455	0.465	0.681	
Hypertension	1.031	0.934–1.138	0.543	0.746	
Myocardial infarction	1.065	0.830–1.368	0.621	0.790	
Dementia	1.106	0.696–1.759	0.669	0.790	
Gastric cancer	0.935	0.674–1.297	0.687	0.790	
Hyperthyroidism	0.929	0.621–1.388	0.718	0.790	
Chronic hepatitis B/C	0.962	0.655–1.414	0.844	0.884	
Other cancer	1.021	0.727–1.433	0.905	0.905	
**B. Covariates.**					
Covariate			Odds Ratio	95% CI	*p*
Pain/discomfort at year t			2.972	2.690–3.285	<0.0001
Age (per SD)			1.664	1.498–1.849	<0.0001
Female			1.487	1.257–1.758	<0.0001
Married			0.966	0.876–1.066	0.493
Household income (log, per SD)			0.908	0.863–0.955	0.000195
Registered disability			1.941	1.629–2.312	<0.0001
Urban residence			0.982	0.891–1.081	0.706
Depressive mood			1.279	1.045–1.566	0.0169
Anxiety			0.964	0.772–1.205	0.749
Suicidal ideation			1.209	0.992–1.473	0.0597
Regular exercise			0.846	0.771–0.929	0.00045
Days walked per week			0.985	0.968–1.003	0.109
Private health insurance			0.967	0.872–1.071	0.52

Generalised estimating equation with binomial family, logit link, exchangeable working correlation and robust standard errors clustered on participant. The model conditions on pain/discomfort and log out-of-pocket payment at year t, includes survey year as a categorical term, and is weighted by the cross-sectional survey weight. All 22 conditions with at least 50 exposed individuals are entered simultaneously in this model. The exposure-specific adjustment sets, which exclude each condition’s established downstream sequelae (Supplementary Table S5), apply to the decomposition models of Table 3 and not to this one. CI, confidence interval; FDR, false discovery rate (Benjamini–Hochberg).

**Table 3:** Counterfactual decomposition of the disease–payment association.

Condition	Cost Ratio (95% CI)	Proportion Mediated, % (95% CI)	Indirect Effect, KRW (95% CI)	q (FDR)
Spine disorders *	1.266 (1.165–1.385)	4.8 (1.1–10.2)	10,644 (2700–20,037) [US$11.6 (2.95–21.9)]	0.19
Osteoarthritis, non-knee *	1.049 (0.937–1.171)	not estimable	8693 (1977–17,297) [US$9.49 (2.16–18.9)]	0.19
Rheumatoid arthritis *	0.916 (0.754–1.123)	not estimable	8190 (954–18,504) [US$8.94 (1.04–20.2)]	0.37
Knee osteoarthritis *	1.222 (1.109–1.344)	4.3 (1.0–10.1)	8075 (1916–15,166) [US$8.82 (2.09–16.6)]	0.19
Asthma *	1.075 (0.872–1.299)	not estimable	6442 (407–15,625) [US$7.03 (0.44–17.1)]	0.39
Angina pectoris *	1.224 (1.070–1.408)	2.8 (0.1–10.6)	5392 (202–13,292) [US$5.89 (0.22–14.5)]	0.39
Depression/bipolar disorder	1.274 (1.052–1.525)	1.8 (−0.4–8.4)	4345 (−886–12,590) [US$4.74 (−0.97–13.7)]	0.61
Cerebral haemorrhage	0.953 (0.739–1.257)	not estimable	4317 (−2700–12,799) [US$4.71 (−2.95–14.0)]	0.61
Chronic kidney disease	1.055 (0.752–1.451)	not estimable	3568 (−4751–17,265) [US$3.90 (−5.19–18.8)]	0.77
Thyroid cancer	1.248 (0.989–1.516)	not estimable	3284 (−3305–14,305) [US$3.59 (−3.61–15.6)]	0.73
Breast cancer	0.868 (0.647–1.137)	not estimable	3192 (−1111–9589) [US$3.48 (−1.21–10.5)]	0.61
Cerebral infarction	1.071 (0.934–1.225)	not estimable	2852 (−1208–9086) [US$3.11 (−1.32–9.92)]	0.61
Hypothyroidism	1.338 (1.115–1.577)	0.6 (−1.4–3.6)	1695 (−3605–8573) [US$1.85 (−3.94–9.36)]	0.79
Myocardial infarction	1.210 (1.046–1.420)	0.5 (−2.4–5.5)	978 (−3360–6600) [US$1.07 (−3.67–7.21)]	0.86
Diabetes mellitus	1.223 (1.131–1.325)	0.5 (−0.7–1.9)	929 (−1163–3506) [US$1.01 (−1.27–3.83)]	0.73
Hypertension	1.071 (0.993–1.161)	not estimable	727 (−671–2736) [US$0.79 (−0.73–2.99)]	0.73
Dementia	0.929 (0.634–1.354)	not estimable	569 (−5899–11,358) [US$0.62 (−6.44–12.4)]	0.94
Other cancer	1.416 (1.126–1.813)	−0.0 (−3.1–2.9)	−60 (−7429–8643) [US$−0.07 (−8.11–9.44)]	0.99
Chronic hepatitis B/C	1.121 (0.782–1.631)	not estimable	−545 (−8897–7248) [US$−0.59 (−9.71–7.91)]	0.94
Hyperthyroidism	1.233 (0.953–1.562)	not estimable	−1403 (−10,863–6121) [US$−1.53 (−11.9–6.68)]	0.86
Gastric cancer	1.118 (0.782–1.577)	not estimable	−1476 (−6685–4716) [US$−1.61 (−7.30–5.15)]	0.79
Colorectal cancer	0.830 (0.649–1.053)	not estimable	−2398 (−8480–2184) [US$−2.62 (−9.26–2.38)]	0.73

Interventional analogues of direct and indirect effects estimated by parametric g-computation with a two-part outcome model, on the scale of expected annual out-of-pocket payment in year *t* + 2. Total = direct + indirect exactly. Confidence intervals are percentile intervals from 2000 participant-level bootstrap replicates in which total, direct and indirect effects were retained jointly; the estimand is defined in Supplementary Text S1. Estimates are weighted by the cross-sectional survey weight and by stabilised inverse-probability-of-censoring weights, with exposure-specific adjustment sets (Supplementary Table S5). All 22 modelled conditions are shown, ordered by the size of the indirect effect, so that no condition is selected for display on the basis of its result. An asterisk marks the six conditions whose indirect-effect confidence interval excludes zero. The *q*-value is the Benjamini–Hochberg false discovery rate value across all 22 modelled conditions, computed from a normal-approximation *p*-value derived from the width of the bootstrap interval (Section 2.6.3); that criterion and the interval need not agree when the bootstrap distribution is skewed. No condition’s indirect effect survives false discovery rate correction (minimum *q* = 0.19); the disease-specific estimates are therefore hypothesis-generating rather than confirmatory. A proportion mediated is reported only where the total effect’s confidence interval excludes zero, which is what permits the ratio to be computed; it does not indicate survival of false discovery rate correction. Supplementary Table S2 additionally reports the total and direct effects in won for every condition.

## Data Availability

The Korea Health Panel Survey data are publicly available from the Korea Health Panel Survey (https://www.khp.re.kr). The analytical code is available from the corresponding author on reasonable request.

## Supplementary Materials

The following supporting information can be downloaded at https://www.mdpi.com/article/10.3390/medicina62081549/s1, Figure S1: directed acyclic graph; Table S1: missing data and censoring by wave; Table S2: full decomposition for all conditions; Table S3: alternative expenditure definition; Table S4: ICD-10 mapping of conditions; Table S5: exposure-specific adjustment sets; Table S6: participants and person-years per condition; Table S7: incident versus prevalent analysis; Table S8: analysis omitting mental-health covariates; Table S9: year-to-year transitions in pain/discomfort; and Text S1: formal estimand and g-computation algorithm.

## Abbreviations

The following abbreviations are used in this manuscript:

CI	Confidence interval
COPD	Chronic obstructive pulmonary disease
COVID-19	Coronavirus disease 2019
CR	Cost ratio
DAG	Directed acyclic graph
EQ-5D	EuroQol five-dimension
FDR	False discovery rate
ICD-10	International Classification of Diseases, 10th Revision
IPCW	Inverse-probability-of-censoring weight
IQR	Interquartile range
IRB	Institutional Review Board
KHPS	Korea Health Panel Survey
KRW	Korean won
OR	Odds ratio
PPP	Purchasing power parity
SD	Standard deviation
SMD	Standardised mean difference
