# Supplementary material for: How Much Chronic Disease Out-of-Pocket Expenditure Runs Through Pain? A Prospective Counterfactual Decomposition of Five-Year Korean Panel Data
Source: Medicina (Kaunas). 2026 Aug 12;62(8):1549. doi: 10.3390/medicina62081549 (PMC13515068; doi:10.3390/medicina62081549)
Supplement: Supplementary file 1 [file medicina-62-01549-s001.zip › Supplementary_Tables_S1-S9.pdf]

Supplementary Materials

How Much Chronic-Disease Out-of-Pocket Expenditure Runs Through Pain? A Prospective Counterfactual Decomposition of Five-Year Korean Panel Data

Table S1. Data availability, censoring and triplet formation by wave

Survey year	Adult person-years	Pain/discomfort observed, %	Payment observed, %	Survey weight available, %	Deaths recorded	Zero-payment person-years, n (%)
2019	12,395	93.5	100.0	100.0	77	2,085 (16.8)
2020	11,424	92.0	100.0	100.0	85	1,601 (14.0)
2021	10,931	92.1	100.0	100.0	75	1,320 (12.1)
2022	10,153	92.3	100.0	100.0	90	836 (8.2)
2023	9,942	90.3	100.0	100.0	78	744 (7.5)

Triplet formation

Baseline year *t*	Eligible person-years	Contributed a complete triplet	% retained
2019	11,274	8,953	79.4
2020	10,316	8,432	81.7
2021	9,901	8,307	83.9

Person-years are restricted to adults aged 19 years or older and censored following a recorded death. A person-year is eligible if exposure, pain/discomfort, payment and all covariates are observed at year *t*; it contributes a triplet if pain/discomfort is also observed at *t*+1 and payment at *t*+2. Censoring is addressed by stabilised inverse-probability-of-censoring weights (Methods, Section 2.5).

Figure S1. Directed acyclic graph underlying the exposure-specific adjustment sets

(See accompanying file FigureS1_DAG.png)

The index chronic disease measured at year *t* is the exposure, pain/discomfort at *t*+1 the intermediate variable, and out-of-pocket payment during *t*+2 the outcome. Demographic, socioeconomic, behavioural and mental-health covariates measured at year *t* are common causes of all three and are adjusted for. Pain/discomfort and payment at year *t* are conditioned on, so the analysis asks whether disease status predicts a change in pain and whether that change predicts subsequent spending.

Conditions that are established downstream sequelae of the index disease lie on the path from exposure to outcome. Adjusting for them would remove part of the association under study and could open a collider path, so each condition's own sequelae are excluded from its adjustment set (Table S5).

Disease severity, treatment intensity and care-seeking propensity are unmeasured. Because they are plausibly affected by the exposure and confound the intermediate–outcome relationship, natural direct and indirect effects are not identified; interventional analogues, which tolerate exposure-induced intermediate–outcome confounding, are estimated instead (Methods, Section 2.6.1). This residual

confounding cannot be eliminated by design and is the principal reason the study is described as exploratory.

Zero-payment person-years are those recording no out-of-pocket payment under the primary expenditure definition (emergency, inpatient, medical outpatient and outpatient pharmacy payments). Across all waves 6,586 of 54,845 adult person-years (12.0%) recorded no payment. The proportion falls monotonically across the study period; person-years with no payment are retained in the analysis and are modelled by the first part of the two-part outcome model.

Table S2. Counterfactual decomposition, all modelled conditions

Condition	Total effect, KRW	Direct, KRW	Indirect, KRW (95% CI)	Cost ratio (95% CI)	Proportion mediated, %	*q* (FDR)
Spine disorders*	221,319 [US$ 242]	210,676 [US$ 230]	10,644 (2,700–20,037) [US$ 11.6 (2.95–21.9)]	1.266 (1.165–1.385)	4.8 (1.1–10.2)	0.19
Osteoarthritis, non-knee*	42,409 [US$ 46.3]	33,715 [US$ 36.8]	8,693 (1,977–17,297) [US$ 9.49 (2.16–18.9)]	1.049 (0.937–1.171)	not estimable	0.19
Rheumatoid arthritis*	−72,732 [US$ −79.4]	−80,922 [US$ −88.3]	8,190 (954–18,504) [US$ 8.94 (1.04–20.2)]	0.916 (0.754–1.123)	not estimable	0.37
Knee osteoarthritis*	187,455 [US$ 205]	179,380 [US$ 196]	8,075 (1,916–15,166) [US$ 8.82 (2.09–16.6)]	1.222 (1.109–1.344)	4.3 (1.0–10.1)	0.19
Asthma*	64,844 [US$ 70.8]	58,402 [US$ 63.8]	6,442 (407–15,625) [US$ 7.03 (0.44–17.1)]	1.075 (0.872–1.299)	not estimable	0.39
Angina pectoris*	192,985 [US$ 211]	187,593 [US$ 205]	5,392 (202–13,292) [US$ 5.89 (0.22–14.5)]	1.224 (1.070–1.408)	2.8 (0.1–10.6)	0.39
Depression / bipolar disorder	235,358 [US$ 257]	231,014 [US$ 252]	4,345 (−886–12,590) [US$ 4.74 (−0.97–13.7)]	1.274 (1.052–1.525)	1.8 (−0.4–8.4)	0.61
Cerebral haemorrhage	−40,320 [US$ −44.0]	−44,637 [US$ −48.7]	4,317 (−2,700–12,799)	0.953 (0.739–1.257)	not estimable	0.61

Condition	Total effect, KRW	Direct, KRW	Indirect, KRW (95% CI)	Cost ratio (95% CI)	Proportion mediated, %	q (FDR)
			[US$ 4.71 (−2.95–14.0)]			
Chronic kidney disease	47,814 [US$ 52.2]	44,246 [US$ 48.3]	3,568 (−4,751–17,265) [US$ 3.90 (−5.19–18.8)]	1.055 (0.752–1.451)	not estimable	0.77
Thyroid cancer	214,356 [US$ 234]	211,071 [US$ 230]	3,284 (−3,305–14,305) [US$ 3.59 (−3.61–15.6)]	1.248 (0.989–1.516)	not estimable	0.73
Breast cancer	−114,701 [US$ −125]	−117,893 [US$ −129]	3,192 (−1,111–9,589) [US$ 3.48 (−1.21–10.5)]	0.868 (0.647–1.137)	not estimable	0.61
Cerebral infarction	60,911 [US$ 66.5]	58,059 [US$ 63.4]	2,852 (−1,208–9,086) [US$ 3.11 (−1.32–9.92)]	1.071 (0.934–1.225)	not estimable	0.61
Hypothyroidism	290,266 [US$ 317]	288,571 [US$ 315]	1,695 (−3,605–8,573) [US$ 1.85 (−3.94–9.36)]	1.338 (1.115–1.577)	0.6 (−1.4–3.6)	0.79
Myocardial infarction	181,245 [US$ 198]	180,267 [US$ 197]	978 (−3,360–6,600) [US$ 1.07 (−3.67–7.21)]	1.210 (1.046–1.420)	0.5 (−2.4–5.5)	0.86
Diabetes mellitus	188,244 [US$ 205]	187,315 [US$ 204]	929 (−1,163–3,506) [US$ 1.01 (−1.27–3.83)]	1.223 (1.131–1.325)	0.5 (−0.7–1.9)	0.73
Hypertension	60,421 [US$ 66.0]	59,694 [US$ 65.2]	727 (−671–2,736) [US$ 0.79 (−0.73–2.99)]	1.071 (0.993–1.161)	not estimable	0.73
Dementia	−61,571 [US$ −67.2]	−62,140 [US$ −67.8]	569 (−5,899–11,358)	0.929 (0.634–1.354)	not estimable	0.94

Condition	Total effect, KRW	Direct, KRW	Indirect, KRW (95% CI)	Cost ratio (95% CI)	Proportion mediated, %	q (FDR)
			[US$ 0.62 (−6.44–12.4)]			
Other cancer	358,172 [US$ 391]	358,231 [US$ 391]	−60 (−7,429–8,643) [US$ −0.07 (−8.11–9.44)]	1.416 (1.126–1.813)	−0.0 (−3.1–2.9)	0.99
Chronic hepatitis B/C	104,613 [US$ 114]	105,158 [US$ 115]	−545 (−8,897–7,248) [US$ −0.59 (−9.71–7.91)]	1.121 (0.782–1.631)	not estimable	0.94
Hyperthyroidism	201,267 [US$ 220]	202,670 [US$ 221]	−1,403 (−10,863–6,121) [US$ −1.53 (−11.9–6.68)]	1.233 (0.953–1.562)	not estimable	0.86
Gastric cancer	101,848 [US$ 111]	103,324 [US$ 113]	−1,476 (−6,685–4,716) [US$ −1.61 (−7.30–5.15)]	1.118 (0.782–1.577)	not estimable	0.79
Colorectal cancer	−146,955 [US$ −160]	−144,557 [US$ −158]	−2,398 (−8,480–2,184) [US$ −2.62 (−9.26–2.38)]	0.830 (0.649–1.053)	not estimable	0.73

* Indirect effect 95% confidence interval excludes zero on a per-condition basis. Confidence intervals are from 2,000 participant-level bootstrap replicates. Estimates are weighted by the cross-sectional survey weight and stabilised inverse-probability-of-censoring weights, with exposure-specific adjustment sets. No indirect effect survives Benjamini–Hochberg false-discovery-rate correction across the 22 conditions (minimum q = 0.19). The proportion mediated is shown only where the confidence interval for the total effect excludes zero. US$ values are purchasing-power-parity equivalents converted at 916.0 KRW per US$ (Section 2.4.1); they are not market-rate conversions.

Table S3. Alternative expenditure definition

Condition	Indirect effect, primary definition	Indirect effect, narrower definition	Difference, %
Spine disorders	10,689*	10,432*	−2.4
Osteoarthritis, non-knee	8,881*	8,539*	−3.9
Rheumatoid arthritis	8,440*	8,222*	−2.6

Condition	Indirect effect, primary definition	Indirect effect, narrower definition	Difference, %
Knee osteoarthritis	8,043*	7,852*	−2.4
Asthma	6,333*	6,231*	−1.6
Angina pectoris	5,342*	5,283*	−1.1
Depression / bipolar disorder	4,379	4,175	−4.7
Cerebral haemorrhage	4,351	4,373	+0.5
Chronic kidney disease	3,553	3,508	−1.3
Thyroid cancer	3,413	3,398	−0.4
Breast cancer	3,146	3,053	−2.9
Cerebral infarction	2,838	2,782	−2.0
Hypothyroidism	1,939	1,858	−4.2
Dementia	1,554	1,509	−2.9
Myocardial infarction	1,148	1,149	+0.1
Diabetes mellitus	868	845	−2.6
Hypertension	729	672	−7.8
Other cancer	412	414	+0.5
Chronic hepatitis B/C	−607	−650	+7.1
Gastric cancer	−1,063	−1,108	+4.2
Hyperthyroidism	−1,309	−1,254	−4.2
Colorectal cancer	−2,676	−2,634	−1.6

* 95% confidence interval excludes zero. Both columns of this table are estimated without censoring weights, so the primary-definition column reproduces the unweighted estimates quoted in Section 3.5 rather than the censoring-weighted estimates of record in Table S2. The primary definition comprises emergency, inpatient and outpatient service payments together with outpatient pharmacy dispensing payments; the narrower definition omits one outpatient component. Dental payments are excluded from both. The same conditions reach significance under either definition.

Table S4. ICD-10 mapping of chronic conditions

Condition as recorded in the Korea Health Panel Survey	Corresponding ICD-10 code(s)
Hypertension	I10–I15
Diabetes mellitus	E10–E14
Chronic hepatitis B/C	B18
Alcoholic hepatitis	K70.1
Liver cirrhosis	K74
Knee osteoarthritis	M17

Condition as recorded in the Korea Health Panel Survey	Corresponding ICD-10 code(s)
Osteoarthritis, non-knee	M15, M16, M18, M19
Rheumatoid arthritis	M05–M06
Gastric cancer	C16
Colorectal cancer	C18–C20
Lung cancer	C34
Breast cancer	C50
Cervical cancer	C53
Thyroid cancer	C73
Other cancer	C00–C97 (other)
Angina pectoris	I20
Myocardial infarction	I21–I22
Cerebral haemorrhage	I60–I62
Cerebral infarction	I63
Asthma	J45–J46
Emphysema	J43
COPD	J44
Bronchiectasis	J47
Hypothyroidism	E03
Hyperthyroidism	E05
Depression / bipolar disorder	F31–F33
Dementia	F00–F03, G30
Chronic kidney disease	N18
Spine disorders	M50–M51, M47–M48

Conditions are self-reported physician diagnoses recorded in the KHPS chronic disease module as ever diagnosed. Codes are indicative mappings provided for international comparability; the survey does not record ICD codes directly. Spine disorders combine intervertebral disc disorder and other spine disease; the survey revised these items in 2021, and although the subcategories are not comparable across that boundary the composite is (Methods, Section 2.4.3).

Table S5. Exposure-specific adjustment sets

Index condition	Conditions excluded from its adjustment set	Rationale
Hypertension	Chronic kidney disease, Cerebral infarction, Cerebral haemorrhage, Myocardial infarction, Angina pectoris, Dementia	Renal, cerebrovascular and ischaemic heart disease are sequelae of hypertension

Index condition	Conditions excluded from its adjustment set	Rationale
Diabetes mellitus	Chronic kidney disease, Cerebral infarction, Cerebral haemorrhage, Myocardial infarction, Angina pectoris, Dementia	Renal, cerebrovascular and ischaemic heart disease are sequelae of diabetes
Chronic hepatitis B/C	Other cancer, Depression / bipolar disorder	Hepatocellular malignancy is a sequela of chronic hepatitis
Alcoholic hepatitis	Liver cirrhosis, Other cancer, Depression / bipolar disorder	Cirrhosis and malignancy are sequelae of alcoholic liver disease
Liver cirrhosis	Other cancer, Depression / bipolar disorder	Hepatocellular malignancy is a sequela of cirrhosis
Knee osteoarthritis	Depression / bipolar disorder	Depression is a recognised consequence of chronic pain
Osteoarthritis, non-knee	Depression / bipolar disorder	Depression is a recognised consequence of chronic pain
Rheumatoid arthritis	Knee osteoarthritis, Osteoarthritis, non-knee, Depression / bipolar disorder	Secondary osteoarthritis and depression are sequelae of rheumatoid arthritis
Gastric cancer	Depression / bipolar disorder	Depression is a recognised consequence of chronic pain
Colorectal cancer	Depression / bipolar disorder	Depression is a recognised consequence of chronic pain
Breast cancer	Depression / bipolar disorder	Depression is a recognised consequence of chronic pain
Thyroid cancer	Hypothyroidism, Hyperthyroidism, Depression / bipolar disorder	Hypothyroidism and hyperthyroidism follow thyroid surgery or ablation
Other cancer	Depression / bipolar disorder	Depression is a recognised consequence of chronic pain
Angina pectoris	Myocardial infarction, Cerebral infarction, Dementia	Myocardial infarction and cerebral infarction are downstream events
Myocardial infarction	Cerebral infarction, Dementia	Cerebral infarction and dementia share downstream vascular pathways
Cerebral haemorrhage	Dementia, Depression / bipolar disorder	Dementia and depression are recognised post-haemorrhage sequelae
Cerebral infarction	Dementia, Depression / bipolar disorder	Dementia and depression are recognised post-stroke sequelae

Index condition	Conditions excluded from its adjustment set	Rationale
Asthma	Depression / bipolar disorder	Depression is a recognised consequence of chronic pain
Hypothyroidism	Depression / bipolar disorder	Depression is a recognised consequence of chronic pain
Hyperthyroidism	Depression / bipolar disorder	Depression is a recognised consequence of chronic pain
Depression / bipolar disorder	Dementia	Dementia is a downstream cognitive outcome
Chronic kidney disease	Myocardial infarction, Angina pectoris, Cerebral infarction, Cerebral haemorrhage	Cardiovascular and cerebrovascular disease are sequelae of chronic kidney disease
Spine disorders	Depression / bipolar disorder	Depression is a recognised consequence of chronic pain

For every other condition, physician-diagnosed depression or bipolar disorder is excluded from the adjustment set on the grounds that it is a recognised consequence of chronic painful disease. A single common adjustment set is not defensible when the conditions are causally related to one another, because adjusting an index condition for its own sequelae removes part of the association under study and may open collider paths. The graph was specified a priori on clinical grounds, before estimation. Each exclusion reflects an established disease-sequela relationship (for example, hypertension and diabetes as causes of chronic kidney disease, stroke and ischaemic heart disease; thyroid surgery or ablation as a cause of post-treatment hypothyroidism; rheumatoid arthritis as a cause of secondary osteoarthritis), and no exclusion was added or removed after inspecting the effect estimates. The directed acyclic graph underlying these sets is shown in Supplementary Figure S1.

Table S6. Participants and person-years per condition

Condition	Individuals ever affected	Person-years affected	Prevalence, %	Individuals in triplet sample	Analytic status
Hypertension	4,624	17,475	33.71	3,411	Modelled
Spine disorders	2,883	9,992	19.27	2,027	Modelled
Knee osteoarthritis	2,399	8,684	16.75	1,786	Modelled
Diabetes mellitus	2,141	7,878	15.20	1,523	Modelled
Osteoarthritis, non-knee	809	2,221	4.28	623	Modelled
Depression / bipolar disorder	577	1,664	3.21	329	Modelled

Condition	Individuals ever affected	Person-years affected	Prevalence, %	Individuals in triplet sample	Analytic status
Cerebral infarction	501	1,666	3.21	315	Modelled
Angina pectoris	468	1,567	3.02	327	Modelled
Myocardial infarction	406	1,336	2.58	300	Modelled
Other cancer	344	992	1.91	193	Modelled
Hypothyroidism	329	1,116	2.15	231	Modelled
Dementia	290	680	1.31	82	Modelled
Asthma	258	851	1.64	176	Modelled
Rheumatoid arthritis	185	589	1.14	132	Modelled
Thyroid cancer	162	609	1.17	121	Modelled
Chronic hepatitis B/C	150	496	0.96	94	Modelled
Hyperthyroidism	148	479	0.92	101	Modelled
Cerebral haemorrhage	144	446	0.86	90	Modelled
Gastric cancer	117	401	0.77	78	Modelled
Breast cancer	117	402	0.78	78	Modelled
Colorectal cancer	115	316	0.61	66	Modelled
Chronic kidney disease	102	302	0.58	61	Modelled
Lung cancer	78	202	0.39	35	Descriptive only
Bronchiectasis	70	205	0.40	49	Descriptive only
COPD	69	202	0.39	46	Descriptive only
Liver cirrhosis	65	206	0.40	41	Descriptive only
Cervical cancer	44	132	0.25	27	Descriptive only
Emphysema	19	62	0.12	16	Descriptive only

Condition	Individuals ever affected	Person-years affected	Prevalence, %	Individuals in triplet sample	Analytic status
Alcoholic hepatitis	18	43	0.08	8	Descriptive only

Conditions with fewer than 50 affected individuals in the triplet sample were not modelled and are reported descriptively only (7 conditions: Alcoholic hepatitis, Liver cirrhosis, Lung cancer, Cervical cancer, Emphysema, COPD, Bronchiectasis). Prevalence is the unweighted proportion of adult person-years with the condition recorded. Because the survey records conditions as ever diagnosed, prevalence accumulates across waves and should not be read as incidence.

Table S7. Incident versus prevalent disease

Condition and disease status	Total effect, KRW	Indirect effect, KRW (95% CI)	Cost ratio (95% CI)
Osteoarthritis, non-knee — prevalent	56,631 [US$ 61.8]	6,862 (−3,143–19,275) [US$ 7.49 (−3.43–21.0)]	1.063 (0.917–1.238)
Spine disorders — prevalent	268,152 [US$ 293]	6,640 (−3,263–17,351) [US$ 7.25 (−3.56–18.9)]	1.309 (1.170–1.486)
Knee osteoarthritis — incident	207,121 [US$ 226]	5,202 (−2,233–16,145) [US$ 5.68 (−2.44–17.6)]	1.235 (1.013–1.519)
Knee osteoarthritis — prevalent	203,486 [US$ 222]	5,005 (−2,290–13,934) [US$ 5.46 (−2.50–15.2)]	1.231 (1.092–1.397)
Angina pectoris — prevalent	188,035 [US$ 205]	4,636 (−2,534–14,503) [US$ 5.06 (−2.77–15.8)]	1.208 (1.024–1.415)
Osteoarthritis, non-knee — incident	54,759 [US$ 59.8]	2,701 (−2,971–13,038) [US$ 2.95 (−3.24–14.2)]	1.061 (0.836–1.301)
Spine disorders — incident	118,476 [US$ 129]	2,689 (−1,474–9,348) [US$ 2.94 (−1.61–10.2)]	1.136 (1.015–1.292)
Cerebral infarction — prevalent	94,418 [US$ 103]	2,190 (−1,622–8,861) [US$ 2.39 (−1.77–9.67)]	1.104 (0.936–1.296)
Hypertension — prevalent	66,415 [US$ 72.5]	611 (−737–2,969) [US$ 0.67 (−0.80–3.24)]	1.075 (0.982–1.185)
Diabetes mellitus — prevalent	230,591 [US$ 252]	309 (−1,540–2,657) [US$ 0.34 (−1.68–2.90)]	1.262 (1.158–1.387)
Hypertension — incident	166,215 [US$ 181]	−898 (−7,149–3,423) [US$ −0.98 (−7.80–3.74)]	1.187 (0.890–1.566)
Angina pectoris — incident	183,411 [US$ 200]	−1,045 (−10,212–10,130) [US$ −1.14 (−11.1–11.1)]	1.203 (0.894–1.636)
Diabetes mellitus — incident	−66,843 [US$ −73.0]	−2,928 (−10,799–2,063) [US$ −3.20 (−11.8–2.25)]	0.924 (0.771–1.130)
Cerebral infarction — incident	163,513 [US$ 179]	−7,957 (−26,248–5,510) [US$ −8.69 (−28.7–6.02)]	1.181 (0.881–1.653)

Incident disease is the first within-person transition from absent to present, identifiable only from 2020; the analysis is restricted to t = 2020 and 2021, splitting each exposure into incident and prevalent strata (16,739 triplets, 8,948 individuals). Confidence intervals are from 2,000 bootstrap replicates. No condition retains a significant indirect effect in either stratum; estimates are directionally consistent with the main analysis but too imprecise to support conclusions, confirming that five waves do not provide sufficient incident cases for pathway analysis.

Table S8. Sensitivity analysis omitting contemporaneous mental-health covariates

Condition	Indirect effect, primary model	Indirect effect, mental-health covariates omitted	Change, %
Spine disorders	10,644 (2,280–18,687)*	11,503 (4,771–19,355)*	+8.1
Osteoarthritis, non-knee	8,693 (1,515–16,027)*	9,542 (3,694–19,090)*	+9.8
Rheumatoid arthritis	8,190 (1,147–16,786)*	8,791 (1,150–17,171)*	+7.3
Knee osteoarthritis	8,075 (1,695–16,072)*	8,419 (2,835–16,275)*	+4.3
Depression / bipolar disorder	4,323 (−646–11,421)	8,040 (1,893–17,884)*	+86.0
Asthma	6,442 (430–13,650)*	7,130 (1,233–17,656)*	+10.7
Angina pectoris	5,392 (585–11,471)*	5,332 (662–13,109)*	−1.1
Cerebral haemorrhage	4,373 (−2,566–13,474)	4,654 (−3,374–13,598)	+6.4
Chronic kidney disease	3,568 (−4,491–17,349)	4,504 (−3,794–19,355)	+26.2
Breast cancer	2,985 (−816–8,117)	3,908 (−1,766–10,650)	+30.9
Thyroid cancer	3,119 (−3,446–17,441)	3,561 (−2,581–16,815)	+14.1
Cerebral infarction	2,852 (−1,413–8,230)	3,309 (−1,302–10,445)	+16.0
Hypothyroidism	1,602 (−3,539–7,007)	2,270 (−2,698–8,670)	+41.6
Dementia	569 (−6,163–16,889)	1,511 (−5,609–13,072)	+165.7
Myocardial infarction	949 (−3,361–6,491)	1,069 (−2,708–6,212)	+12.7
Diabetes mellitus	929 (−1,264–3,016)	955 (−1,174–3,846)	+2.8
Hypertension	727 (−687–2,629)	727 (−689–2,682)	+0.0
Other cancer	−30 (−6,781–9,235)	601 (−9,128–8,017)	not meaningful
Chronic hepatitis B/C	−553 (−8,923–8,767)	−564 (−7,665–7,503)	+2.0
Gastric cancer	−1,345 (−6,652–3,415)	−1,218 (−7,192–6,066)	−9.5
Hyperthyroidism	−1,351 (−10,733–7,108)	−1,465 (−12,539–6,356)	+8.5
Colorectal cancer	−2,376 (−8,677–2,277)	−2,900 (−9,184–2,032)	+22.1

* 95% confidence interval excludes zero. Depressive mood, anxiety and suicidal ideation in the past year are measured contemporaneously with the exposure and may lie on the pathway under study rather than confound it. Omitting them increases indirect effects for most conditions, and one further condition — depression or bipolar disorder — has an interval excluding zero that does not in the primary model, which is consistent with partial mediation through mental health. Primary estimates are therefore conservative in this respect. Both columns of this table were estimated for the purpose of this comparison,

so that they may be read against each other. The primary estimates of record are those of Table S2. For the six conditions whose indirect-effect interval excludes zero in the primary analysis, the point estimates in the primary-model column above are identical to those of Table S2; for the remaining conditions they differ by at most 207 KRW, and no condition changes its status. Confidence intervals in this table derive from a separate bootstrap and are therefore not numerically identical to those of Table S2.

Table S9. Year-to-year transitions in pain/discomfort

Wave pair	Pairs observed	Persisted (1 →1)	Resolved (1 →0)	New onset (0→1)	Remained absent (0→0)	Resolved among those with pain, %
2019→2020	10,049	2,110	1,413	1,389	5,137	40.1
2020→2021	9,473	1,868	1,411	1,147	5,047	43.0
2021→2022	8,954	1,797	1,035	1,323	4,799	36.5
2022→2023	8,649	1,806	1,204	1,118	4,521	40.0
All pairs	37,125	7,581	5,063	4,977	19,504	40.0

Transitions are computed over all consecutive wave pairs for participants observed in both waves. Among participants reporting pain/discomfort in a given wave, 40.0% no longer reported it one year later, and only 60.0% reported it in both waves. Because the EQ-5D item refers to the survey day and waves are one year apart, pain that arises and resolves entirely within an inter-wave interval is unobserved, and pain present at the exposure wave but resolved by the outcome wave does not contribute to the prospective association. This transience is the basis for regarding the prospective cost ratio (Section 3.3) as biased downwards, undercounting short-lived pain, complementary to the contemporaneous estimate, which is biased upwards by simultaneity; the two are expected to be biased in opposite directions.

Text S1. Formal estimand and g-computation algorithm

Let A denote the index chronic condition at year t (1 = present, 0 = absent), M the binary pain/discomfort indicator at year t+1, Y the out-of-pocket payment during year t+2, and C the vector of year-t covariates including prior pain and prior log payment. Let $M(a)$ be the potential mediator under exposure a and $Y(a,m)$ the potential outcome under exposure a and mediator m.

We target randomised-interventional analogues of the direct and indirect effects. Let $G(a)$ be a random draw from the distribution of $M(a)$ given C, drawn independently of the natural mediator value. The overall effect is decomposed as

- interventional direct effect: E[Y(1, G(0))] − E[Y(0, G(0))]
- interventional indirect effect: E[Y(1, G(1))] − E[Y(1, G(0))]

so that their sum equals the total effect E[Y(1, G(1))] − E[Y(0, G(0))]. Unlike the natural (pure/total) direct and indirect effects, these quantities do not require cross-world independence and remain identified when mediator–outcome confounders are affected by the exposure.

Under consistency, positivity, and no-unmeasured-confounding for the exposure–mediator, exposure–outcome and mediator–outcome relationships given C, each term is identified by the g-formula

$E[Y(a, G(a^*))] = \Sigma\_c \, \Sigma\_m \, E[Y \mid A=a, M=m, C=c] \cdot P(M=m \mid A=a^*, C=c) \cdot P(C=c)$.

Estimation proceeds by: (i) fitting $P(M \mid A, C)$ by logistic regression; (ii) fitting $E[Y \mid A, M, C]$ as a two-part model — logistic for $P(Y>0 \mid A, M, C)$ and gamma with log link for $E[Y \mid Y>0, A, M, C]$ — with expected payment $E[Y \mid A, M, C] = P(Y>0 \mid \cdot) \cdot E[Y \mid Y>0, \cdot]$; (iii) for each participant, summing the fitted $E[Y \mid A=a, M=m, C]$ over $m \in \{0,1\}$ weighted by the fitted $P(M=m \mid A=a,$ *C), which evaluates the inner sum analytically and avoids Monte-Carlo error; and (iv) averaging over the empirical distribution of* C^* with cross-sectional survey weights and stabilised inverse-probability-of-censoring weights. The exposure of interest *A* varies across the 22 models; all other conditions enter *C* according to the exposure-specific adjustment set (Table S5). Confidence intervals are percentile intervals from 2,000 participant-level bootstrap resamples, with total, direct and indirect effects retained jointly on each replicate so that intervals for the cost ratio and the proportion mediated follow directly.
